# Professionals’ perceptions of complexity and preferences for interprofessional collaborative practice in pediatric rehabilitation: Insights from social network analysis

**DOI:** 10.1177/18758894261502416

**Published:** 2026-11-11

**Authors:** Lisa-Maria van Klaveren, Rien de Vos, Vincent Geukers, Marjolijn Ketelaar, Jan Willem Gorter, Mattijs Alsem

**Affiliations:** 1 Institute for Education and Training, Amsterdam UMC, Location University of Amsterdam, Amsterdam, The Netherlands; 2 Amsterdam Public Health Research Institute, Quality of Care, Amsterdam, The Netherlands; 3 Department of Pediatric Intensive Care, Emma Children’s Hospital, Amsterdam UMC, Amsterdam, The Netherlands; 4 Center of Excellence for Rehabilitation Medicine, UMC Utrecht Brain Center, University Medical Center Utrecht and De Hoogstraat Rehabilitation, Utrecht, The Netherlands; 5 Department of Rehabilitation, Physical Therapy Science & Sports, UMC Utrecht Brain Center, University Medical Center Utrecht, Location Wilhelmina Children’s Hospital, Utrecht, The Netherlands; 6 CanChild, Department of Pediatrics, 3710 McMaster University, Hamilton, ON, Canada

**Keywords:** complexity, interprofessional collaborative practice, social network analysis, integrated care, conjoint methodology

## Abstract

**Purpose:**

Integrated care for children with disability and complex care needs requires professionals to collaborate across various disciplines and settings. However, it remains uncertain how professionals interpret complexity and how these interpretations influence composition and collaboration in integrated care networks. This study aimed to investigate professionals’ perceptions of complexity and their preferences for interprofessional collaborative practice.

**Method:**

This cross-sectional study using a web-based survey with scripted cases included 120 professionals working in pediatric rehabilitation medicine across the Netherlands. Scripted cases were created using the ICF-framework, selecting two elements per domain and modeling each domain at three levels of difficulties (low, medium, high). Thirteen scripted cases were randomly selected using conjoint methodology. For each case, professionals’ 1) perceived case complexity and 2a) preferred interprofessional composition were recorded. Social network analysis was applied to examine 2b) preferred collaborative practice per case using four integrated care network metrics: density, overall span (diameter), mean distance between members, and cohesiveness (transitivity).

**Results:**

Higher levels of difficulties in each ICF-domain significantly increased professionals’ perceptions of complexity. Preferences for interprofessional composition varied according to levels of domain difficulties and exhibited substantial inter-individual variation. Although higher perceived case complexity was generally associated with preferences for greater cohesiveness, shorter mean distances, and more compact and denser networks, domain-specific difficulties also influenced preferred interprofessional collaborative practices even when overall perceived case complexity was low.

**Conclusions:**

The study identified that preferences for interprofessional collaborative practice varied in relation to perceived case complexity and among professionals. Recognizing these differences at network-level is vital, as it enables the effective functioning of these networks and contributes to improved integrated care for children with disability.

## Introduction

Children with disability constitute a growing population within pediatric care.^1–3^ Their care needs typically span cognitive, emotional, physical, and psychosocial domains,^4–6^ often rendering their care inherently complex and surpassing the capabilities of any single profession.^7^ As a result, integrated care requires interprofessional collaborative practice that actively engages these children, their families, and professionals from various disciplines, including allied health (e.g., physical therapists, occupational therapists, dietitians), medical (e.g., general pediatricians, rehabilitation physicians, neurologists), nursing (e.g., pain management or feeding nurse specialists), and psychosocial professionals (e.g., psychologists, social workers).^8,9^ In pediatric rehabilitation, interprofessional teams are commonly tasked with supporting children with disability and complex care needs, guiding, sustaining or improving their functioning and participation throughout their development.^10,11^ To ensure quality and continuity of care on individual and population levels, these teams increasingly work within integrated care networks.^12,13^

These networks typically comprise professionals from various disciplines across a diverse range of settings, such as primary healthcare, academic centers of expertise, and public or municipal services, including child welfare and family support.^14^ They are often coordinated by (rehabilitation) physicians, designated care coordinators or case managers, who are responsible for integrating care across professions and settings, in alignment with the goals, needs, and preferences of children and their families.^12,15,16^ While previous research suggests that integrated care networks can enhance knowledge sharing, skill development, care accessibility, and communication, the lack of a shared understanding regarding who should collaborate, when, and how poses a significant barrier to their effectiveness. As a result, this often leaves parents with the burden of relaying information and bridging gaps between professionals and settings.^13,17–22^

According to Reeves and colleagues,^23,24^ the way professionals collaborate to integrate care effectively is closely linked to the complexity of care situations. They identify four types of interprofessional collaborative practice ‒ “teamworking,” “collaborating,” “coordinating,” and “networking” ‒ which differ in the extent to which members are interdependent, have clear roles and goals, experience integration, share responsibility, and develop a team identity.^25^ In pediatric rehabilitation, the management of complex care may necessitate “teamworking” in that members heavily rely on each other’s expertise. When complexity decreases, less intensive practices may suffice, such as “collaborating” or “coordinating” in which goals and responsibilities are more distributed across the integrated care network.^22,23^ In the context of low-complexity or routine care, loosely connected “networking” may be more suitable. This practice is characterized by minimal interdependence, weak integration, and limited coordination needs.

Although conceptually well-founded, it remains uncertain how individual professionals interpret care situations and how these interpretations influence their preferences for interprofessional composition and collaborative practice, let alone how ideally composed networks with shared collaborative practice emerge.^26^ These different perspectives may also vary depending on professional background (e.g., medical, allied health, psychosocial) and setting (e.g., hospital, community, school), leading to significant variation in preferences and expectations of interprofessional collaborative practice.^27–29^ Such insights can guide the design and implementation of integrated care networks, tailored to the diverse perspectives of professionals and families. This may contribute to achieving the quintuple aim of healthcare improvements: enhancing patient and family experience, improving population health, reducing costs of service utilization, increasing professionals’ satisfaction, and promoting health equity.^30,31^

### Objectives

The primary objective was twofold. First, the study aimed to examine the impact of difficulties in domains, using scripted cases developed based on the International Classification of Functioning, Disability and Health (ICF) framework, on a) perceptions of complexity and b) preferences for interprofessional collaborative practice, specifically regarding preferred interprofessional composition across professions and settings. The second was to assess the association between perceptions of complexity and preferences for interprofessional collaborative practice, as measured by four network metrics: density, diameter, mean distance, and transitivity. The secondary objective was to explore whether perceptions and preferences differed between and within professional groups, particularly comparing medical specialists (e.g., rehabilitation physicians) and allied health or psychosocial professionals.

## Methods

### Design and setting

This study employed a cross-sectional design, using a web-based survey of scripted cases to better understand professionals’ perceptions of complexity and preferences for interprofessional collaborative practice in pediatric rehabilitation. The study was conducted in the Netherlands, where pediatric rehabilitation care is organized across multiple settings in a tiered system. In this system, allied health and psychosocial professionals operate in primary, secondary, or tertiary care settings. Primary care professionals, such as general practitioners, physical therapists or social workers, typically work in private practices or community-based settings and are accessible without referral. Secondary care involves general pediatrics and more specialized rehabilitation services delivered by multidisciplinary teams based in rehabilitation centers or hospitals. For particularly complex or rare conditions, children may receive tertiary care, often provided in academic or highly specialized hospital settings. Data was collected between February and December 2024.

Eligible participants for this study were professionals working in rehabilitation practice in primary, secondary, or tertiary care settings. This included medical specialists (e.g., pediatricians, pediatric rehabilitation physicians), allied health professionals (e.g., physical, occupational or speech therapists), and psychosocial professionals (e.g., psychologists, social workers).

### Procedure

Professionals working in pediatric rehabilitation across the Netherlands were invited through convenience sampling via emails and social media posts (i.e., LinkedIn). They completed a web-based survey in Qualtrics (Provo, UT), which presented a series of scripted cases. Upon accessing the survey, participants first received an information letter, provided informed consent, and reviewed an example case before proceeding. Each participant was presented with the same scripted cases in a random order. Following each case, participants answered two questions designed to assess their perceptions of complexity and preferences for interprofessional collaborative practice. Upon completion of the final case, participants responded to a brief set of demographic questions, including items on their professional background, years of experience, training status, and care setting (i.e., primary, secondary, or tertiary care). Following survey completion, participants received a summary of their responses and were offered the option to participate in a follow-up conversation for additional clarification or discussion. Field notes were taken during these conversations which were not recorded. The English translation of the Dutch, web-based survey can be found in the Supplemental materials.

### Case descriptions

To systematically model case complexity, conjoint analysis was employed. This methodology is widely used in both economics and healthcare to assess how individuals value specific attributes within a decision-making context.^32–34^ To ensure comparability and contextual relevance, a baseline scenario was developed, serving as a standardized reference for all scripted cases. Case descriptions were operationalized using the ICF, which is widely used in pediatric rehabilitation for describing and structuring human functioning.^35,36^ Originally introduced as a classification system, the ICF has evolved into a comprehensive conceptual model for rehabilitation goals and practices encompassing five interrelated domains: body functions/anatomical structures, activities, participation, personal factors, and environmental factors.^35^ Two representative elements were selected to represent each domain. To assess the impact of domains on perceptions and preferences, three ordinal levels of difficulties were defined for each domain. These levels reflected the low, medium, and high difficulties in the two selected elements across the extent of impairment in body functions and structures, the degree of limitations in activities and of restrictions in participation, as well as the changing influence of environmental and personal factors (see Table 1, Figure 1).

**Table 1.** Choice of ICF-domains with two elements and three levels of difficulties each.

ICF-domain	Elements	Level of difficulties per domain		
		Low	Medium	High
Body functions & anatomical structures (BDY)	Sensory functions and pain, movement-related functions	Mild shoulder and neck pain, arm movement not restricted	Moderate shoulder and neck pain, arm movement partly restricted	Severe shoulder and neck pain, arm movement fully restricted
	Skin and related structures	Head wound healing well	Head wound leaking	Head wound with pus formation
Activities (ACT)	Self-care	Enough energy for ADLs	Low energy for ADLs	No energy ADLs
	Communication	Answers questions in full sentences	Answers questions with yes/no	Does not answer questions
Participation (PAR)	Leisure activities	Plays basketball as part of a team, participates in matches	Plays basketball individually, cannot participate in matches	Recently cut from the U16 basketball team
	Education	No school absenteeism	Excused school absenteeism (15% of school time)	Unexcused school absenteeism (30% of school time)
Personal factors (PER)	Physical disability	Recovered from a broken ankle two years ago	Lower leg amputation due to trauma six years ago	Complete spinal cord injury (L2) due to bleeding four years ago
	Character	Known as a cheerful, active teenager	Known as a restless, impulsive teenager	Known as a difficult teenager with autistic behaviors
Environmental factors (ENV)	Insurance status	Supplementary health insurance	Basic health insurance	No health insurance
	Family support	Parents speak good Dutch/English	Single parent speaks limited Dutch/English	Parents in conflictual divorce, do not speak Dutch/English

*Note.* Each scripted case combined one level of domain difficulties (low, medium or high) for each ICF-domain.

ICF: International Classification of Functioning, Disability and Health. ADL: activity of daily living.

**Figure 1.**
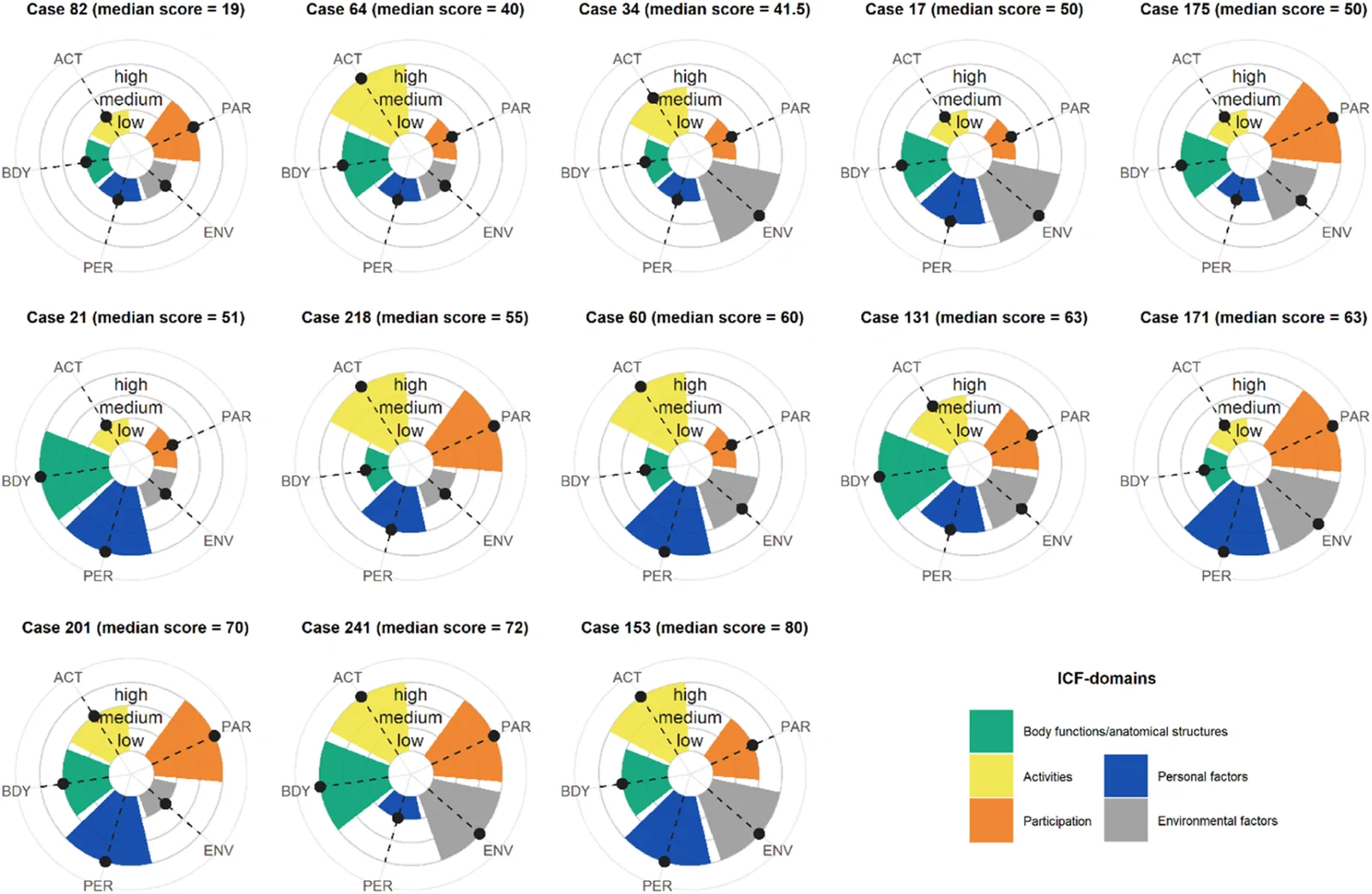
Overview of the randomly selected 13 scripted cases, ordered by median score for perceived case complexity. Note. Case numbers refer to the randomly selected cases from the total number of 243 (35^5^) possible cases based on the five ICF-domains with three levels of domain difficulties each. BDY: Body functions & anatomical structures; ACT: Activities; PAR: Participation; PER: Personal factors; ENV: Environmental factors; ICF: International Classification of Functioning, Disability and Health.

The selection and operationalization of elements and their levels of difficulties were conducted by two authors (MA, LK) based on clinical expertise and methodological considerations. Specifically, the elements and levels were required to meet three criteria: a) relevance to baseline situation, b) meaningfulness and distinguishability for participants, and c) feasibility in terms of the total number of cases required for the analysis. While the ICF employs five qualifiers (0 = no problem, 1 = mild, 2 = moderate, 3 = severe, 4 = complete problem), a three-level system was adopted in this study to maintain practical relevance to the baseline scenario, ensure clear discriminability for participants, and optimize analytical feasibility. Consequently, there was no direct mapping between the standard ICF-qualifiers and the chosen levels of domain difficulties.

To model the effects of five ICF-domains (each with three levels of domain difficulties) on the outcome variables, a full-factorial design would require participants to evaluate 243 (3^5^) cases. However, due to feasibility constraints, 13 cases were randomly selected using the conjoint package in R (seed = 123). This set of cases was evaluated by all participants. Systematically varying domain difficulties across cases allowed estimation of the relative importance of each domain (main effects) while minimizing participant burden. The trade-off was that interaction effects between domains could not be reliably estimated with a fractional factorial design, as implemented here.

### Measurement

To develop a multidimensional understanding of professionals’ perceptions of complexity and preferences for interprofessional collaborative practice, including differences between professional groups (allied health/psychosocial professional vs. medical specialist), three variables were distinguished: perceived case complexity, preferred interprofessional composition, and preferred collaborative practice per case (see Table 2). To contextualize the survey findings, field notes from follow-up conversations were reviewed to provide additional insights.

**Table 2.** Overview of outcome variables with operationalization and scale for perception of complexity and preferences for interprofessional collaborative practice.

Outcome variable	Operationalization	Scale
***Perception of complexity***		
Perceived case complexity	How do you evaluate the situation of the patient described at this moment?	Very simple (0) to Very complex (100)
***Preference for interprofessional collaborative practice***		
Below you will find a list of potential team members for the patient described. In what way should the desired team members collaborate with each other (in writing, through consultation, or in an MDTM)? *One answer per team member is possible. Please select the most ideal option.*		
*Inclusion of professions*		
Medical specialists	General practitioner, pediatric rehabilitation physician, neurologist, orthopedist, another medical specialist	No contact (0)
		Written (0)
		Consultation (1)
		MDTM (1)
Allied health professionals	Occupational therapist, physical therapist, nurse, speech therapist, other allied health professional	No contact (0)
		Written (0)
		Consultation (1)
		MDTM (1)
Psychosocial professionals	Social worker, pedagogical specialist, psychologist, mental health nurse practitioner	No contact (0)
		Written (0)
		Consultation (1)
		MDTM (1)
*Inclusion of settings*		
Primary care	General practitioner, occupational therapist, physical therapist, nurse, mental health nurse practitioner, social worker, another primary professional	No contact (0)
		Written (0)
		Consultation (1)
		MDTM (1)
Specialized rehabilitation services	Pediatric rehabilitation doctor, occupational therapist, physical therapist, speech and language therapist, psychologist, social worker, other rehabilitation professional	No contact (0)
		Written (0)
		Consultation (1)
		MDTM (1)
Other setting	Youth mental health worker, child protection agent, school professional, neurologist, orthopedist, professional in another setting	No contact (0)
		Written (0)
		Consultation (1)
		MDTM (1)

*Note.* Respondents could select 0 to 20 team members to be included per case. Connections were calculated for each pair of included team members, ranging from 1 dyadic connection (when 2 team members were included) to 190 dyadic connections (when all team members are included).

*MDTM:* multidisciplinary team meeting.

#### Perceptions of complexity

To examine perceived case complexity, participants scored the case complexity on a continuous scale from very simple (0) to very complex (100), with score of 50 being neither simple nor complex. For instance, if a participant selected 77 on this scale, then their response was recorded as a perceived case complexity score of 77.

#### Preferences for interprofessional collaborative practice

To examine professionals’ preferences for interprofessional collaborative practice, participants were asked to indicate which professionals should be included and in what way members should collaborate (Table 2).

#### Interprofessional composition

For the inclusion of professions, a score of 1 was given when at least one professional within each professional group (allied health professionals, medical specialists, psychosocial professionals) was selected as “in consultation” or “present during multidisciplinary team meeting (MDTM).” Otherwise, it was coded with 0. For the inclusion of settings, a score of 1 was given when at least one professional within each setting (primary care, rehabilitation care, other setting) was selected as “in consultation” or “present during MDTM.” Otherwise, it was coded with 0. For example, if a participant selected the general practitioner, physical therapist, and social worker in primary care, as well as the pediatric rehabilitation doctor and physical therapist in specialized rehabilitation services for consultation or an MDTM, their response would be coded as medical specialists = 1, allied health professionals = 1, and psychosocial professionals = 1 for profession, and primary care = 1, specialized rehabilitation services = 1, and other settings = 0 for setting.

#### Collaborative practice

To examine preferred collaborative practice within integrated care networks, social network analysis was employed using the R packages *igraph*, *ggraph*, and *tidygraph*.^37^ Social network analysis examines the structures of relationships and interdependencies among individuals.^38^ It models relationships as multivariate dependent variables, where individual connections are analyzed alongside network statistics and covariates like characteristics such as profession or setting. A social network consists of nodes in terms of profession and setting (e.g., a physical therapist in primary care, a psychologist in specialized rehabilitation services, or a neurologist in other settings) connected by ties, with dyads (pairs) and triads (triples) often serving as key units of analysis.^38^ These analyses were conducted on group level using weighted co-selection matrices, in which edge weights reflected the frequency with which professionals were jointly selected for each case, corrected for the number of entries per case. For instance, if 60% of participants selected both the mental health nurse practitioner and the school setting, and 40% of the participants who selected the mental health nurse practitioner also selected the psychologist in primary care, while none of these participants selected the neurologist, then the resulting edge weights would be mental health nurse practitioner – school = .60; mental health nurse practitioner – psychologist = .40; mental health nurse practitioner – neurologist = 0; school – neurologist = 0; and psychologist – neurologist = 0. Four network metrics were calculated to characterize preferred collaborative practice within the integrated care network for each case using all available responses.

Network density (the proportion of observed connections relative to all possible connections, weighted by strength/frequency) reflects the breadth of interactions. Lower density aligns with a preference for “networking,” where connections are sparse and distributed, while higher density suggests more frequent exchanges, a precursor to more “collaborating.”

Diameter (the maximum shortest path length between any two professionals, using transformed weights) measures network compactness. A smaller diameter indicates a more efficient structure where information flows quickly, characteristic of a preference for “coordinating,” a practice that bridges “networking” and “collaboration” by organizing interactions systematically.

Mean distance (the average shortest path length between all reachable pairs) captures accessibility. Shorter distances imply a more integrated network, reflecting a preference for “collaborating,” where professionals actively work together across domains and settings, moving beyond loose ties toward integrated action.

Transitivity (the global clustering coefficient, calculated as the ratio of closed triplets to all connected triplets) quantifies team cohesion. High transitivity indicates tightly knit subgroups, corresponding to a preference for “teamworking,” where professionals within clusters collaborate closely and consistently, representing the highest level of integration in the contingency approach.

All networks were visualized using *ggraph* with layouts based on the Fruchterman-Reingold algorithm.^39^ Node attributes, such as profession (e.g., allied health, medical, psychosocial) and setting (e.g., primary care, specialized rehabilitation setting, other), were incorporated.

### Statistical methods

To describe the sociodemographic characteristics of the sample, frequencies for professional background, setting and training status, and median and interquartile range (IQR) for work experience were computed. Perceived case complexity and the inclusion of professions and settings were summarized for each case using median and IQRs and frequencies with percentages.

#### Perception of complexity

To verify whether the scripted cases successfully varied in perceived case complexity, a Kruskal-Wallis test was conducted to compare median perceived case complexity across cases among all participants. To examine the individual effects of ICF-domains on perceived case complexity, linear mixed-effects models were employed using the *lme4* package in R.^40^ The five ICF-domains were included as fixed-effect predictors, along with professional group (allied health/psychosocial professionals as reference category). Dummy coding was used to represent the three levels of domain difficulties within each ICF-domain, with low difficulties serving as reference category. Perceived complexity was treated as a continuous dependent variable, and a linear model with Gaussian error distribution and identity link function was specified.

To account for the repeated-measures design, individual participants were modeled as random intercepts. Model estimation was performed using restricted maximum likelihood (REML), and bobyqa optimizer was specified in the control settings to improve convergence. Participants who did not complete all case-based questions were excluded from the analysis to ensure a complete dataset and valid within-subject comparisons. Statistical significance was determined using an alpha level of *p* ≤ .05.

#### Preference for interprofessional collaborative practice

##### Interprofessional composition

To examine the individual effects of ICF-domains on the inclusion of professions (medical, allied health, psychosocial) or setting (primary, specialized rehabilitation, other), generalized linear mixed-effects models were employed using the *lme4* package in R.^40^ The five ICF-domains were entered as fixed-effect predictors, along with professional group (with allied health/psychosocial professional as reference category). For each domain, dummy coding was applied to represent three degrees of complexity, with low complexity serving as reference category. Inclusion of professions and settings were each treated as dichotomous dependent variables, modeled using logistic regression with a binomial distribution and a logit link function.

To account for the repeated-measures design, individual participants were included as random intercepts. Models were estimated using maximum likelihood with Laplace approximation, and bobyqa optimizer was specified to improve convergence. For ease of interpretation, regression coefficients were exponentiated to obtain odds ratios, and the intercept was transformed to a baseline probability using the logistic function (*p* = 1/[1 + exp(−β_0_)]). The baseline probability describes the likelihood of a profession or setting to be included in the integrated care network if all predictors were set to the reference category (ICF-domain: low difficulties; professional group: allied health/psychosocial professional). Participants who did not complete all case-based questions were excluded from the analysis to ensure a complete dataset and valid within-subject comparisons. Statistical significance was determined using an alpha level of *p* ≤ .05.

##### Collaborative practice

To assess the associations between perceived case complexity and network metrics, four one-sided Pearson product-moment correlation coefficients were computed between median perceived case complexity and each network metric: transitivity (positive association), mean distance (negative association), density (negative association), and diameter (positive association). To account for non-normal distributions, correlations were calculated using rank-transformed scores. Statistical significance was determined using an alpha level of *p* ≤ .05.

## Results

### Participants

A total of 120 professionals participated in the study. Of these, 56 (46.7%) participants completed all 13 case-based questions, while the remaining participants provided partial responses with 12 (10%) completing at least seven cases and 52 (43.3%) answering six or fewer cases. The number of responses per case ranged between 67 and 76 (*median* = 71, *IQR* = 2). The sample included 19 pediatric rehabilitation physicians, 17 physical therapists, six occupational therapists, five psychologists, three speech and language therapists, three social therapists, two physician assistants, and one pedagogical specialist.

Forty-six professionals worked in a rehabilitation center, 17 in a hospital setting (12 in academic and six in non-academic hospitals), and two in private practices. The median work experience was 17 years (*IQR* = 15.5). One professional was in training. Sixty-four participants did not report their professional background or setting. Eight participants (two pediatric rehabilitation physicians, two speech therapists, one physician assistant, one physical therapist, one psychologist, and one social therapist) participated in follow-up interviews aimed at elaborating on their decision-making processes.

### Perception of complexity

Perceived case complexity varied across cases (χ^212^ = 362.77, *p* < .001) (see Table 3 for descriptive statistics per case). All ICF-domains had a significant impact on perceived case complexity. From low to high levels of domain difficulties, personal factors had the strongest effect (*B* = 17.17, *SE* = 1.12, *t*(662) = 15.39, *p* < .001, 95% CI [14.99, 19.34]), followed by body functions/anatomical structures (*B* = 14.11, *SE* = 1.32, *t*(662) = 10.72, *p* < .001, 95% CI [11.54, 16.67]), participation (*B* = 14.09, *SE* = 1.12, *t*(662) = 12.63, *p* < .001, 95% CI [11.91, 16.26]), and environmental factors (*B* = 13.39, *SE* = 1.12, *t*(662) = 12.01, *p* < .001, 95% CI [11.22, 15.57]). The impact of domain difficulties on perceived case complexity did not differ between allied health/psychosocial professionals and medical specialists (*B* = −4.20, *SE* = 3.78, *t*(54) = −1.11, *p* = .271, 95% CI [−11.59, 3.20]) (see Figure 2). However, the model indicated substantial residual variance in perceived case complexity (*σ*^*2*^ = 165.70, *SD* = 12.87). The marginal R^2^ indicated that the fixed effects accounted for 38.3% of the variance, whereas the conditional R^2^ showed that the full model explained 68.4% of the variance.

**Table 3.** Descriptive statistics for perceived case complexity and the inclusion of professions (medical specialist, allied health professionals, psychosocial professions) and settings (primary, specialized rehabilitation, other settings) for each case, ordered by median score for perceived case complexity.

Case	n	Perceived case complexity Median (IQR)	Inclusion of professions			Inclusion of settings		
			Medical specialist # Incl. (%)	Allied health prof # Incl. (%)	Psycho- social prof # Incl. (%)	Primary care # Incl. (%)	Spec. rehab. # Incl. (%)	Other settings # Incl. (%)
82	76	19 (18.5)	43 (56.6)	37 (48.7)	15 (19.7)	43 (56.6)	24 (31.6)	7 (9.2)
64	71	40 (30)	53 (74.7)	58 (81.7)	44 (62.0)	44 (612.0)	40 (56.3)	45 (63.4)
34	67	41.5 (22.3)	56 (83.6)	62 (92.5)	23 (34.3)	51 (76.1)	34 (50.8)	33 (49.3)
17	72	50 (31)	64 (88.9)	69 (95.8)	31 (43.1)	52 (72.2)	51 (70.8)	25 (34.7)
175	72	50 (32.8)	59 (81.9)	66 (91.7)	47 (65.3)	57 (79.2)	44 (61.1)	29 (40.3)
21	72	51 (32.5)	49 (68.1)	63 (87.5)	35 (48.6)	41 (56.9)	48 (66.7)	24 (33.3)
218	70	55 (30)	52 (74.3)	48 (68.6)	58 (82.9)	49 (70.0)	39 (55.7)	48 (68.6)
60	67	60 (26.8)	54 (80.6)	62 (92.5)	47 (70.2)	45 (67.2)	46 (68.7)	49 (73.1)
131	75	63 (24.8)	70 (93.3)	70 (93.3)	60 (80.0)	46 (61.3)	61 (81.3)	52 (69.3)
171	70	63 (30.3)	61 (87.1)	65 (92.9)	55 (78.6)	52 (74.3)	52 (74.3)	33 (44.3)
201	70	70 (25)	56 (80.0)	60 (85.7)	55 (78.6)	38 (54.3)	47 (67.1)	41 (58.6)
241	74	72 (23)	73 (98.7)	70 (94.6)	69 (93.2)	53 (71.6)	61 (82.4)	59 (79.7)
153	71	80 (21.3)	68 (95.8)	68 (95.8)	63 (88.7)	47 (66.2)	64 (90.1)	56 (78.9)

*Note.* n = number of participants per case. # Yes represents the frequency with which a profession or setting was included in the integrated care network through consultation or multidisciplinary team meeting.

IQR: interquartile range; Prof: professional; Spec. rehab.: specialized rehabilitation; Incl.: included.

Field notes from follow-up conversations highlighted professionals’ perspectives on complexity as a multidimensional construct, spanning motor skills, behavior, and family dynamics. Emotional and social challenges, language barriers, and school-related issues were frequently noted as contributing factors. In contrast, medical or physical problems were often viewed as less complex when considered in isolation. Additionally, the families’ ability to learn and perform medical tasks was identified as a key influence on professionals’ assessments of complexity.

**Figure 2.**
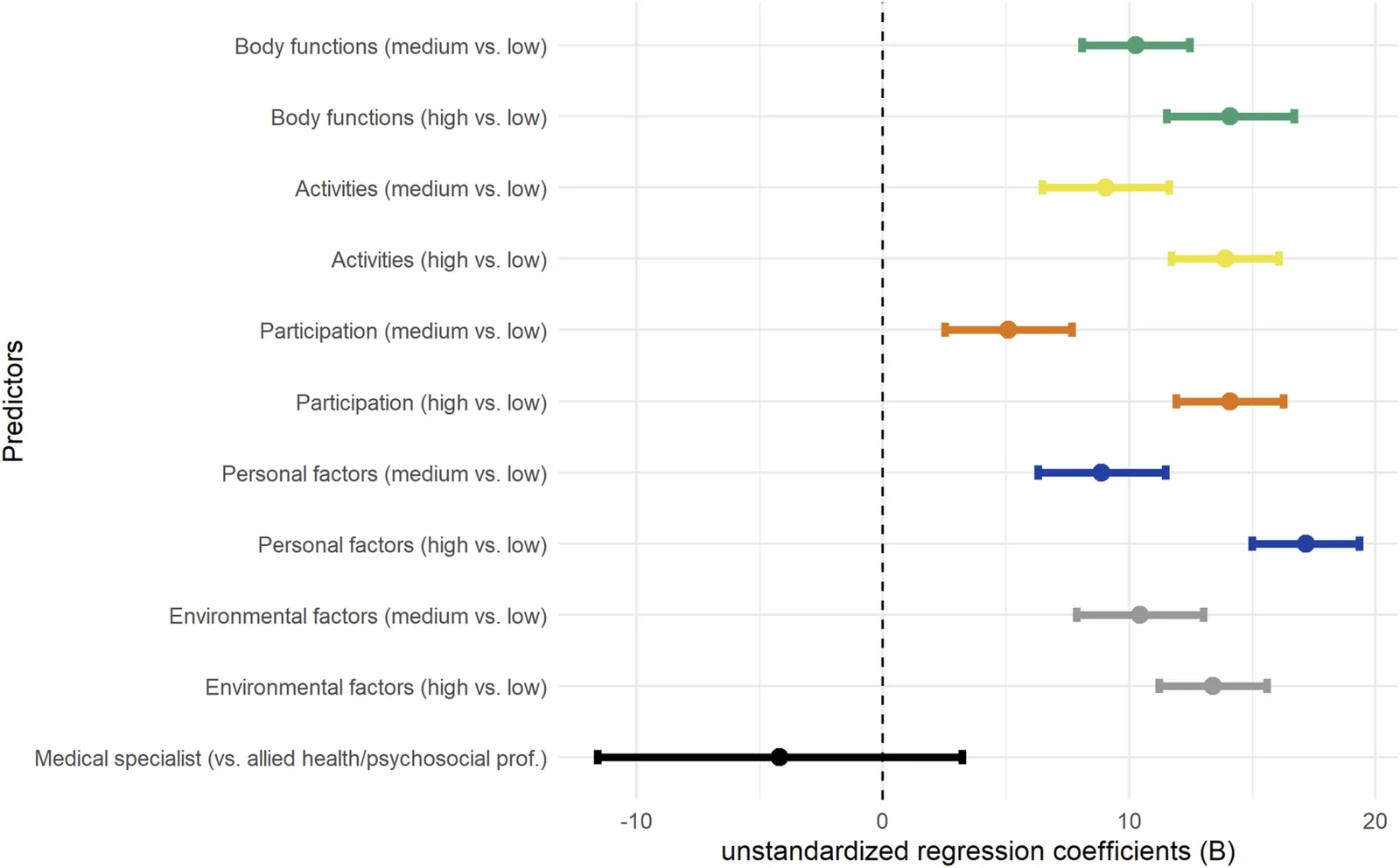
Results of the linear mixed-effects model predicting perceived case complexity across five ICF-domains (reference: low difficulties) and professional group (reference: allied health/psychosocial professional), with random intercepts for individual participants (n = 728 observations nested within 56 participants). Note: Points represent unstandardized regression coefficients (B), and horizontal lines indicate 95% confidence intervals. Positive values indicate higher outcome scores relative to the reference category. ICF: International Classification of Functioning, Disability and Health; prof: professional.

### Preferences for interprofessional collaborative practice

#### Interprofessional composition

##### Inclusion of professions

The baseline probability for at least one medical specialist to be included in the integrated care network was .47 (95% CI [.22, .73]). Medical specialists were more likely to be included when domain difficulties were high compared to low, except for personal factors (see Table 4 *Medical specialists*). There were no statistically significant differences between professional groups. However, substantial heterogeneity in preferences was observed across individual participants (*σ*^*2*^ = 3.29, *SD* = 1.81). The model explained 24.1% of the variance at the fixed-effects level and 65.6% including random effects.

**Table 4.** Results of the logistic mixed-effects model predicting the inclusion of professions across five ICF-domains (reference: low difficulties) and professional group (reference: allied health/psychosocial professional), with random intercepts for individual participants (n = 728 observations nested within 56 participants).

Term	Medical specialists			Allied health professionals			Psychosocial professionals		
	OR	p	95%CI	OR	p	95%CI	OR	p	95%CI
Body function (medium vs. low)	**2.87**	**0.001**	**[1.51, 5.46]**	**2.92**	**0.009**	**[1.31, 6.52]**	**3.00**	**<0.001**	**[1.73, 5.19]**
Body function (high vs. low)	**4.28**	**0.002**	**[1.69, 10.87]**	**2.66**	**0.040**	**[1.04, 6.79]**	**5.91**	**<0.001**	**[2.67, 13.09]**
Activities (medium vs. low)	1.88	0.068	[0.95, 3.69]	1.64	0.294	[0.65, 4.15]	**2.57**	**0.002**	**[1.43, 4.62]**
Activities (high vs. low)	**4.05**	**<0.001**	**[1.87, 8.79]**	1.42	0.398	[0.63, 3.18]	**18.31**	**<0.001**	**[9.20, 36.45]**
Participation (medium vs. low)	2.36	0.062	[0.96, 5.78]	***0.34***	***0.020***	***[0.14, 0.84]***	**3.51**	**0.001**	**[1.72, 7.17]**
Participation (high vs. low)	**2.34**	**0.014**	**[1.19, 4.61]**	0.66	0.310	[0.30, 1.47]	**13.28**	**<0.001**	**[7.12, 24.74]**
Personal factors (medium vs. low)	0.91	0.808	[0.41, 1.99]	1.73	0.256	[0.67, 4.48]	1.55	0.179	[0.82, 2.92]
Personal factors (high vs. low)	0.99	0.983	[0.50, 1.96]	2.08	0.072	[0.94, 4.59]	**2.77**	**0.001**	**[1.56, 4.93]**
Environmental factors (medium vs. low)	**3.12**	**0.001**	**[1.63, 5.96]**	**14.56**	**<0.001**	**[5.15, 41.11]**	**2.38**	**0.004**	**[1.32, 4.28]**
Environmental factors (high vs. low)	**13.96**	**<0.001**	**[6.06, 32.14]**	**17.14**	**<0.001**	**[6.94, 42.35]**	**3.40**	**<0.001**	**[1.79, 6.45]**
Medical specialist (vs. allied health/psychosocial professional)	0.64	0.498	[0.18, 2.33]	***0.16***	***0.012***	***[0.04, 0.69]***	***0.11***	***0.001***	***[0.03, 0.41]***

*Note.* OR = odds ratio. CI = confidence interval. Bold formatting indicate a significant increase (p ≤ .05). Bold italic formatting indicate a significant decrease (p ≤ .05).

The baseline probability for at least one allied health professional to be included in the integrated care network was high (*p* = .82, 95% CI [.57, .94]). The likelihood increased with high (vs. low) difficulties in the environmental factor and body functions/anatomical structures domains (see Table 4 *Allied health professionals*). Medical specialists were generally less likely to include allied health professionals than therapists and psychologists. Again, substantial inter-individual differences were observed (*σ*^*2*^ = 4.67, *SD* = 2.16). The model explained 28.1% of the variance at the fixed-effects level and 70.3% including random effects.

The baseline probability for at least one psychosocial professional to be included in the integrated care network was low (*p* = .07, 95% CI [.03, .18]). All five ICF-domains had a significant impact on the likelihood of their inclusion (see Table 4 *Psychosocial professionals*). In general, medical specialists were less likely than allied health/psychosocial professionals to include psychosocial professionals. Again, substantial heterogeneity in preferences was observed across individual participants (*σ*^*2*^ = 4.28, *SD* = 2.07). The model explained 47.6% of the variance at the fixed-effects level and 73.8% including random effects.

Field notes from follow-up conversations suggested that rehabilitation physicians played a central role as gatekeepers, responsible for shaping and coordinating the integrated care network based on their pre-assessment. Allied health and psychosocial professionals noted that they often consulted peers within their own professions to address specific care needs. For example, speech and language therapists frequently communicated with physical therapists and dieticians, while social workers and psychologists collaborated closely across overlapping areas of expertise. In addition to routine MDTMs in specialized rehabilitation settings, professionals participated in domain-specific meetings, such as psychosocial team meetings (e.g., nurses, psychologists, social therapists, rehabilitation physicians) or allied health meetings (e.g., physical therapists, occupational therapists, rehabilitation physicians). Both physical therapists and psychologists also acknowledged the interdependence of their work; psychologists noted that their contributions created the conditions necessary for physical therapists to focus on physical improvement.

##### Inclusion of settings

The baseline probability for at least one professional working in primary care to be included in the integrated care network was high (*p* = .73, 95% CI [.55, .85]). The likelihood of their inclusion increased when difficulties in the environmental factor were medium and high (see Table 5 *Primary care setting*). There were no differences between allied health/psychosocial professionals and medical specialists. Like the preferences for professional integration, inter-individual differences in the preferred inclusion of primary care settings (*σ*^*2*^ = 2.62, *SD* = 1.62) were observed. The model explained 5.9% of the variance at the fixed-effects level and 47.6% including random effects.

**Table 5.** Results of the logistic mixed-effects model predicting the inclusion of settings across five ICF-domains (reference: low difficulties) and professional group (reference: allied health/psychosocial professional), with random intercepts for individual participants (n = 728 observations nested within 56 participants).

Term	Primary care setting			Specialized pediatric rehabilitation			Other setting		
	OR	p	95% CI	OR	p	95% CI	OR	p	95% CI
Body function (medium vs. low)	0.93	0.751	[0.61, 1.43]	**3.57**	**<0.001**	**[2.13, 5.98]**	**1.76**	**0.016**	**[1.11, 2.77]**
Body function (high vs. low)	0.64	0.074	[0.39, 1.04]	**6.69**	**<0.001**	**[3.50, 12.81]**	**2.00**	**0.019**	**[1.12, 3.58]**
Activities (medium vs. low)	0.83	0.450	[0.50, 1.36]	1.18	0.559	[0.68, 2.06]	**6.68**	**<0.001**	**[3.87, 11.53]**
Activities (high vs. low)	0.89	0.596	[0.58, 1.36]	**3.10**	**<0.001**	**[1.81, 5.31]**	**20.82**	**<0.001**	**[12.04, 36.02]**
Participation (medium vs. low)	0.71	0.169	[0.44, 1.16]	1.86	0.073	[0.94, 3.67]	0.71	0.273	[0.39, 1.30]
Participation (high vs. low)	1.21	0.375	[0.79, 1.85]	**1.74**	**0.026**	**[1.07, 2.83]**	**1.88**	**0.006**	**[1.20, 2.93]**
Personal factors (medium vs. low)	0.96	0.860	[0.58, 1.58]	**2.11**	**<0.001**	**[1.18, 3.77]**	1.52	0.123	[0.89, 2.59]
Personal factors (high vs. low)	0.69	0.078	[0.45, 1.04]	**3.79**	**0.001**	**[2.23, 6.46]**	**1.83**	**0.011**	**[1.15, 2.92]**
Environmental factors (medium vs. low)	**2.92**	**<0.001**	**[1.74, 4.90]**	**2.72**	**<0.001**	**[1.55, 4.79]**	**2.63**	**0.001**	**[1.52, 4.55]**
Environmental factors (high vs. low)	**2.27**	**<0.001**	**[1.49, 3.46]**	**5.75**	**0.012**	**[3.30, 10.02]**	**2.52**	**<0.001**	**[1.56, 4.06]**
Medical specialist (vs. allied health/psychosocial professional)	0.56	0.253	[0.21, 1.51]	***0.25***	***0.049***	***[0.06, 1.00]***	0.71	0.541	[0.24, 2.12]

*Note.* OR = odds ratio. CI = confidence interval. Bold formatting indicate a significant increase (p ≤ .05). Bold italic formatting indicate a significant decrease (p ≤ .05).

The baseline probability for at least one professional working in specialized rehabilitation to be included in the integrated care network was low (*p* = .19, 95% CI [.08, .40]). All five ICF-domains had a significant impact on the likelihood of their inclusion (see Table 5 *Specialized rehabilitation setting*). In general, medical specialists were less likely than therapists/psychologists to integrate specialized rehabilitation professionals within the integrated care networks. However, there were substantial inter-individual differences (*σ*^*2*^ = 5.26, *SD* = 2.29). The model explained 21.4% of the variance at the fixed-effects level and 69.8% including random effects.

The baseline probability for at least one professional working in another setting to be included in the integrated care network was low (*p* = .06, 95% CI [.03, .15]). The likelihood of their inclusion was also significantly impacted by all five ICF-domains (see Table 5 *Other setting*). There were no differences between allied health/psychosocial professionals and medical specialists. However, substantial inter-individual differences in preferences were observed (*σ*^*2*^ = 3.28, *SD* = 1.81). The model explained 26.3% of the variance at the fixed-effects level and 63.1% including random effects.

Field notes from follow-up conversations suggested the critical role of specialized rehabilitation settings, where professionals noted functions such as diagnostics, initial screening, and ongoing guidance and evaluation within primary care environments. Certain medical conditions, such as newly acquired paraplegia, and specific interventions, like splinting, often required treatment in specialized settings. Professionals indicated that coordination across these settings typically occurred within domains. For example, allied health professionals in specialized rehabilitation frequently collaborated with primary care colleagues, while psychosocial professionals linked with schools, and rehabilitation physicians coordinated with general practitioners or other medical specialists. Field notes also revealed distinctions between different types of meetings: routine team meetings within specialized settings, standardized cross-setting meetings organized through care pathways, and ad hoc meetings across settings as needed. Additionally, professionals in rural areas noted that treatment decisions, particularly in primary care, were shaped by practical factors, including travel distance and service availability.

#### Collaborative practice

Integrated care networks showed different preferred collaborative practices based on perceived case complexity (see Figure 3). More specifically, more complex cases were associated with preferences for more densely connected (*density*: *r* = .92, *t*^11^ = 7.83, *p* < .001, 95% CI [.79, 1.00]), more compact (*diameter*: *r* = –.60, t^11^ = –2.50, *p* = .014, 95% CI [–1.00, –.17]), and efficiently connected networks with shorter distances between members *(mean distance: r* = –.77, *t*^11^ = –4.02, *p* = .001, 95% CI [–1.00, –.46]). Moreover, increased perceived case complexity was related to preferences for more cohesive teams within the integrated care network (*transitivity*: *r* = .81, *t*^11^ = 4.50, *p* < .001, 95% CI [.53, 1.00]). Case 64 (high difficulties in activity, medium difficulties in body functions and anatomical structures) appeared to deviate from the linear relationship, exhibiting a preference for smaller diameter and shorter distances between members than would be expected based on perceived case complexity. Similarly, preferred cohesiveness in case 82 (medium difficulties in participation) was higher than expected based on its low perceived case complexity.

**Figure 3.**
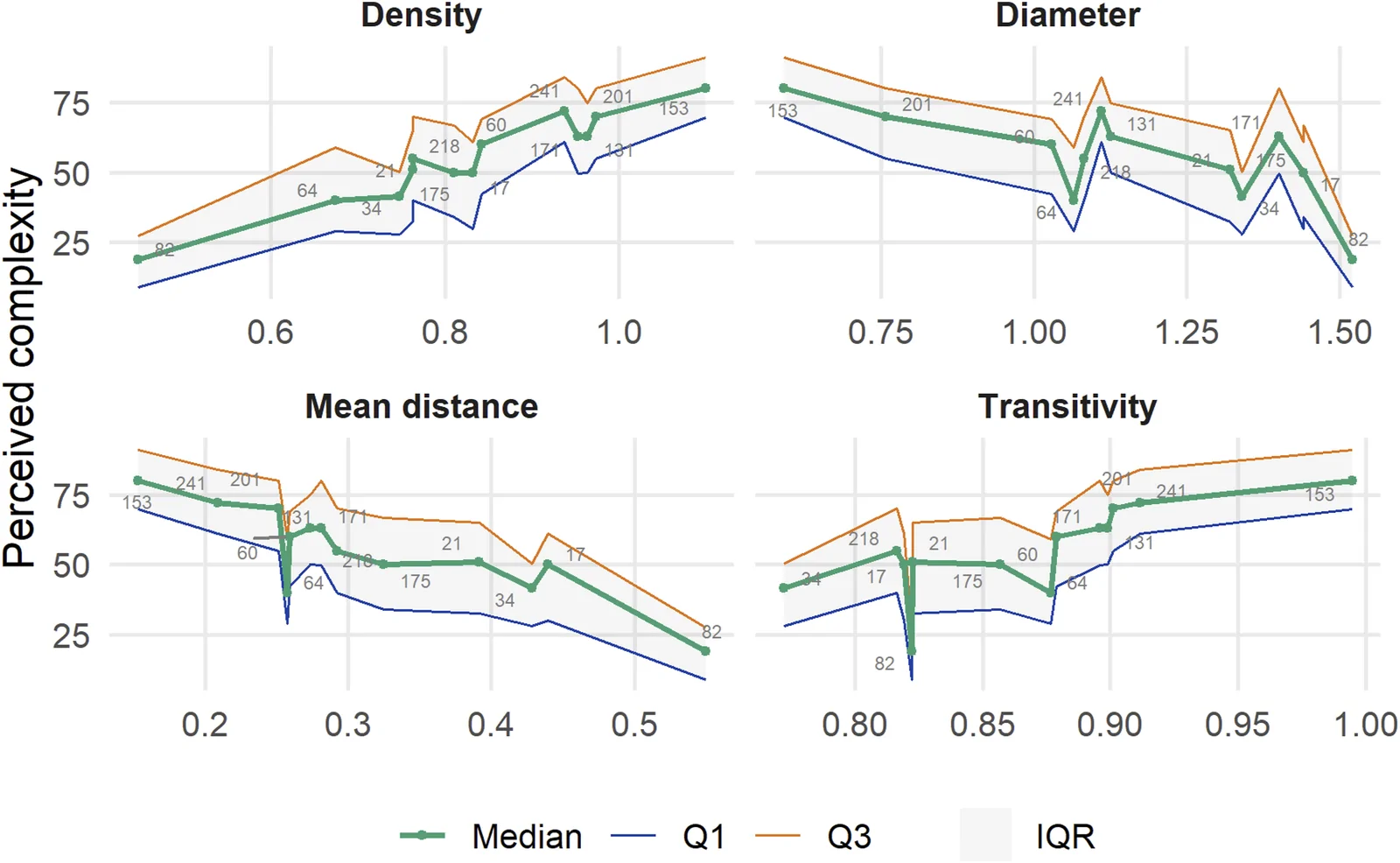
Correlations between median perceived case complexity (with IQR) and network metrics (density, diameter, mean distance and transitivity). Note: IQR: interquartile range.

Additionally, participants’ preferences for the distribution of professions and settings, as well as the preferred distances between them within integrated care networks, varied by case complexity (see Figure 4). In less complex cases, participants preferred clustering by setting, particularly within primary and specialized rehabilitation services (e.g., triangles clustered around x=.5/y=1 or rectangles around x=1/y=.5 in case 64), with rehabilitation physicians identified as the key link between settings (e.g., orange rectangle positioned centrally around x=.5/y=.5 between clusters in case 82). In contrast, for more complex cases, participants preferred inclusion of professionals across multiple settings, with rehabilitation physicians and schools occupying central roles (e.g., the orange rectangle and green circle positioned centrally in cases 131 and 241). Other settings, such as child protection or mental healthcare, were perceived as peripheral (e.g., grey nodes at the edges in cases 17 and 241) or excluded entirely (e.g., no grey nodes in case 82) when perceived case complexity was low.

**Figure 4.**
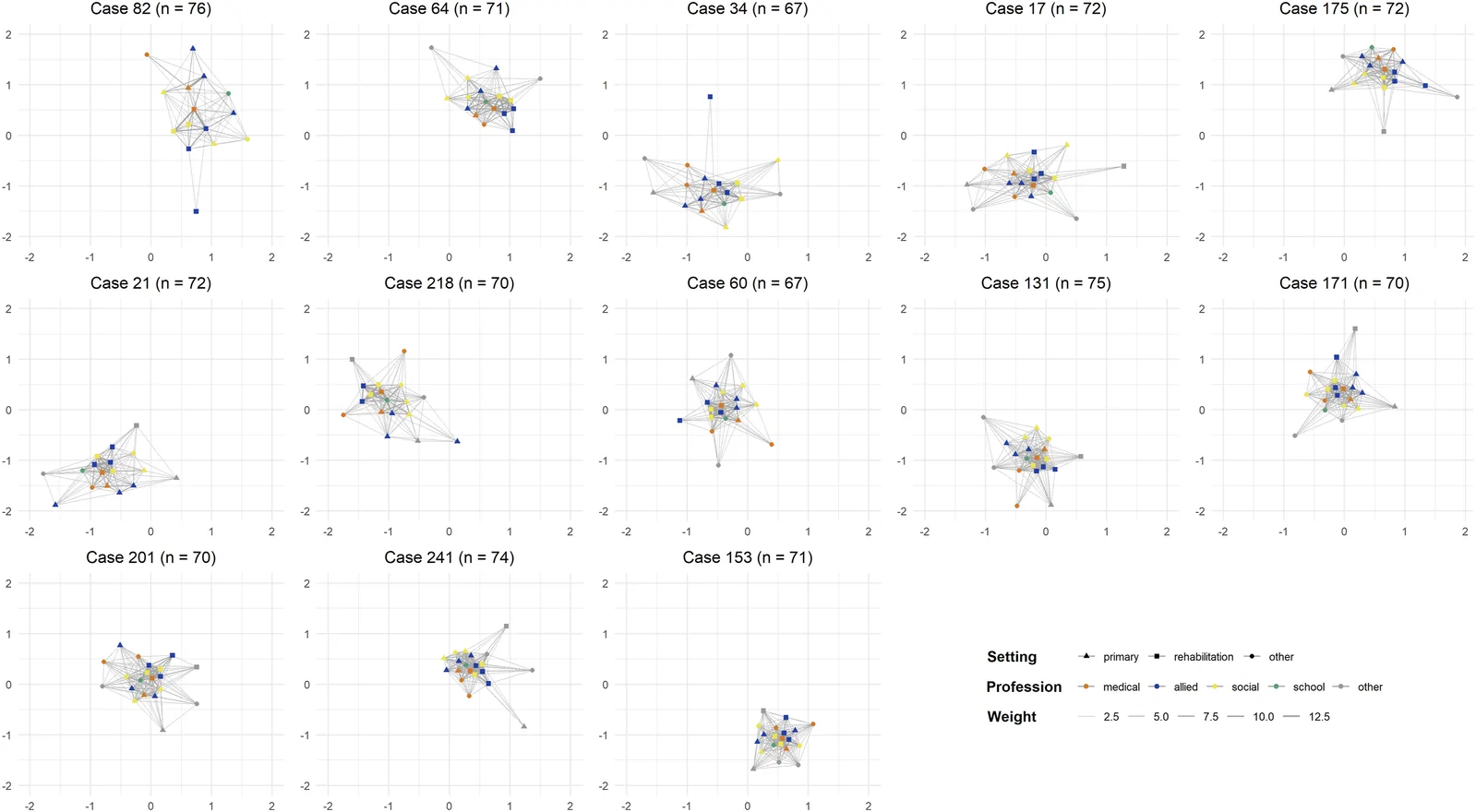
Integrated care network by case (number of participants per case), ordered by median score for perceived case complexity. Note: The x- and y-axes are arbitrary and reflect the spatial arrangement generated by the Fruchterman-Reingold algorithm, which positions nodes to minimize edge crossings and group well-connected nodes. The spatial arrangement is held constant across integrated care networks to ensure comparability.

## Discussion

This study offers new insights into how professionals perceive complexity in scripted cases and envision the interprofessional composition and collaborative practices within integrated care networks in pediatric rehabilitation. First, professionals considered all five ICF-domains to contribute to the complexity of individual cases, with slightly greater emphasis on “personal factors” and “participation.” This balanced focus may reflect the typically holistic orientation of pediatric rehabilitation, in contrast to acute hospital settings where clinical attention often centers on “body functions and anatomical structures”.^41^ Contrary to previous studies reporting differences in complexity perception between medical specialists and nurses,^41^ no significant differences between medical specialists and allied health/psychosocial professionals were observed in this study. This may be attributable to the long-standing practice of embedded interprofessional teams in Dutch rehabilitation settings, which could foster shared cognition.^42^ Nonetheless, notable variation in perceived case complexity was observed among individuals, regardless of professional group. These findings suggested that perceptions of complexity are neither solely shaped by ICF-domain characteristics nor professional background. Instead, other influences, such as prior experiences, personal norms and values, specific work environments, and exposure to different care situations, likely also shape these perceptions.

Second, regarding the preferences for interprofessional collaborative practice, the findings indicated that allied health professionals and medical specialists were included in the integrated care network by default, particularly when complexity in “environmental factors” and “body functions and anatomical structures” was high. In contrast, the inclusion of psychosocial professionals, who are typically not standard members of the integrated care network (as reflected by their low baseline probability), was primarily influenced by complexity in “activity” and “participation” domains. These distinctions may reflect varying scopes of practice and domain-specific expertise among professionals. For example, medical specialists and allied health professionals may share a functional scope characterized by interdependent expertise, whereas the scope of psychosocial professionals tends to be more focused on social or behavioral aspects, with their expertise generally perceived as more independent. Moreover, allied health and psychosocial professionals demonstrated a greater tendency than rehabilitation physicians to include non-medical colleagues. This may be linked to a shared and general understanding of the role of rehabilitation physicians, while allied health and psychosocial professionals have more insights into each other’s domain-specific and interdependent expertise and actionable contributions. Additionally, this may also underline the role of rehabilitation physicians as gatekeepers who shape and coordinate the integrated care network, while, once involved, allied and psychosocial professionals seek the full breadth of interprofessional integration in the network.

Patterns related to the inclusion of settings showed that “environmental” complexity influenced professionals’ preferences for collaboration between primary, specialized rehabilitation and other settings. Such collaboration may be necessary to address situations in which families lack sufficient resources to manage care needs effectively. Conversely, complexity in “activities” prompted the preferred inclusion of professionals in other settings such as schools. This trend may be linked to the recognized role that schools play in the development of children with disability and the goal of mitigating the impact of energy and behavioral constraints on school performance. Rehabilitation physicians appeared to more closely gatekeep the necessity for specialized rehabilitation settings, as evidenced by their lower tendency to include specialized settings with a low baseline probability compared to primary care settings. Nonetheless, considerable inter-individual variation was observed, which was not directly related to professional background, along with unexplained variance. This suggests the potential influence of additional factors as well as domain interactions that warrant further exploration.

Third, the findings revealed that perceived case complexity of cases was related to professionals’ preferred collaborative practice in integrated care networks. Specifically, higher perceived case complexity was linked to a preference for denser, more compact networks with shorter distances between members. This may reflect a shift from the preferred collaborative practice of “networking,” more typical in simpler cases where members are more dispersed, towards smaller yet denser integrated care networks that emphasize “coordinating” and “collaborating.” Furthermore, higher perceived case complexity was associated with a preference for greater transitivity, or team cohesiveness, within integrated care networks. This suggests a more tightly connected approach to “teamworking” across the integrated care network. These observations align with the contingency approach proposed by Reeves and colleagues,^23,24^ which posits that interprofessional collaborative practice should adapt to situational demands. In highly complex cases, intensive “teamworking” characterized by shared goals, interdependence, and collective action appears essential, whereas routine cases can be adequately managed through more loosely connected “networking.” Together, these findings point towards preferences for collaborative practices that respond flexibly to changing clinical contexts and the needs of patients and their families, underscoring the importance incorporating dynamic systems principles.^15,43,44^

Interestingly, the findings suggested deviations from a strictly linear relationship between perceived case complexity and network metrics, indicating avenues for further investigation. Future studies could explore why cases with certain domain-specific needs, despite not being perceived as complex, tended to elicit preferences for the formation of smaller, closely connected teams. Additionally, these studies should employ ICF-qualifiers to align with standard outcome measures and assessment tools in clinical research and practice, thereby enhancing transferability. A more nuanced understanding of the relationship between complexity and interprofessional collaborative practice can inform the design and implementation of integrated care strategies. Adapting interprofessional collaborative practices to situational constraints may also reduce the burden on parents, who often serve as intermediaries by relaying information and bridging gaps between professionals and care settings.

### Limitations

Several limitations must be acknowledged. First, while the use of scripted, paper-based cases and the conjoint method aids in facilitating standardized comparisons, they may not fully correspond with the nuanced and dynamic nature of real-life clinical decision-making. However, there are limited alternative statistical methods available to quantify these complex decision-making processes. While the selection of two elements per ICF-domain and three levels of domain difficulties was manageable for participants, it constrained the spectrum of complexity captured. Complexity may not be the sum of domain-specific difficulties; instead, it may emerge from their dynamic and interdependent interplay. For example, a “medium” limitation in activities might result in minimal participation restrictions in a highly supportive environment, whereas the same limitation could lead to greater restrictions in a resource-limited setting, thereby increasing complexity. Thus, complexity in pediatric rehabilitation may be best described as qualitative properties or differences arising from these interplays, which inform care possibilities for children with disability. In contrast, disability itself reflects the relational product of a person within their environment. While this systemic relationship is inherently complex and dynamic, it does not necessarily translate to complexity in pediatric rehabilitation care.

Additionally, reliance on self-reported data introduces potential biases, such as social desirability or discrepancies between perceived and actual behavior. To better understand how professionals perceive and act upon complexity and integrated care networks in practice, future research should triangulate qualitative data from interviews, longitudinal observations, and real-time data, such as network tracking or electronic health record analysis. Furthermore, the care question or goal was not clearly articulated, which allowed for exploration of various functions of specialized rehabilitation settings but may have impacted how participants interpreted complexity and formulated preferences for interprofessional composition. While this study intentionally focused on professionals’ direct interactions without the reliance of families as intermediaries, their exclusion reduces the completeness of the network. Lastly, while the sample included various professional groups, most participants were from specialized rehabilitation settings which limits the generalizability of findings to primary or community care contexts.

Despite these limitations, the key strength of this study lies in its systematic application and quantitative analysis of decision-making using valid statistical techniques. This approach facilitates a controlled and replicable modeling of perceptions of multidimensional complexity and preferences for interprofessional collaborative practice in terms of interprofessional composition and collaborative practices. By modeling key elements across five ICF-domains within a consistent clinical context, this study effectively explored how functional, activity, participatory, personal, and environmental factors shape professionals’ perceptions and preferences.

## Supplemental material

Supplemental material - Professionals’ perceptions of complexity and preferences for interprofessional collaborative practice in pediatric rehabilitation: Insights from social network analysis Supplemental material for Professionals’ perceptions of complexity and preferences for interprofessional collaborative practice in pediatric rehabilitation: Insights from social network analysis by Lisa-Maria van Klaveren, Rien de Vos, Vincent Geukers, Marjolijn Ketelaar, Jan Willem Gorter, Mattijs Alsem in Journal of Pediatric Rehabilitation Medicine.

## Data Availability

The data that support the findings of this study are not openly available due to reasons of sensitivity and are available from the corresponding author upon reasonable request. Data are located in controlled access data storage at UMC Utrecht.*
