# Supplementary material for: Professionals’ perceptions of complexity and preferences for interprofessional collaborative practice in pediatric rehabilitation: Insights from social network analysis
Source: J Pediatr Rehabil Med. 2026 Nov 11:18758894261502416. Online ahead of print. doi: 10.1177/18758894261502416 (PMC13768321; doi:10.1177/18758894261502416)
Supplement: Supplementary file 1 [file sj-pdf-1-prm-10.1177_18758894261502416.pdf]

English version of the web-based survey

Cases

Case 82

Patient (15 years old), known at the community or rehabilitation center where you work, fell down the stairs a week ago. The head wound was sutured. The patient was referred to a neurologist for further examination. No abnormalities were found on the Computed Tomography (CT) scan.
Today, the patient presents again at the center.

**Body functions**	Mild shoulder and neck pain, arm movement not restricted
	Head wound healing well
**Activities**	Enough energy for daily activities (ADLs)
	Answers questions in full sentences
**Participation**	Plays basketball individually, cannot participate in matches
	Excused school absenteeism (15% of school time)
**Personal factors**	Recovered from a broken ankle 2 years ago
	Known as a cheerful, active teenager
**Environmental factors**	Supplementary health insurance
	Parents speak good Dutch/English

Case 34

Patient (15 years old), known at the community or rehabilitation center where you work, fell down the stairs a week ago. The head wound was sutured. The patient was referred to a neurologist for further examination. No abnormalities were found on the Computed Tomography (CT) scan.
Today, the patient presents again at the center.

**Body functions**	Mild shoulder and neck pain, arm movement not restricted
	Head wound healing well
**Activities**	Low energy for daily activities (ADLs)
	Answers questions with yes/no
**Participation**	Plays basketball as part of a team, participates in matches
	No school absenteeism
**Personal factors**	Recovered from a broken ankle 2 years ago
	Known as a cheerful, active teenager
**Environmental factors**	No health insurance
	Parents in conflictual divorce, do not speak Dutch/English

Case 64

Patient (15 years old), known at the community or rehabilitation center where you work, fell down the stairs a week ago. The head wound was sutured. The patient was referred to a neurologist for further examination. No abnormalities were found on the Computed Tomography (CT) scan.
Today, the patient presents again at the center.

**Body functions**	Moderate shoulder and neck pain, arm movement partly restricted
	Head wound leaking
**Activities**	No energy for daily activities (ADLs)
	Does not answer questions
**Participation**	Plays basketball as part of a team, participates in matches

	No school absenteeism
**Personal factors**	Recovered from a broken ankle 2 years ago
	Known as a cheerful, active teenager
**Environmental factors**	Supplementary health insurance
	Parents speak good Dutch/English

Case 17

Patient (15 years old), known at the community or rehabilitation center where you work, fell down the stairs a week ago. The head wound was sutured. The patient was referred to a neurologist for further examination. No abnormalities were found on the Computed Tomography (CT) scan.<br>Today, the patient presents again at the center.

**Body functions**	Moderate shoulder and neck pain, arm movement partly restricted
	Head wound leaking
**Activities**	Enough energy for daily activities (ADLs)
	Answers questions in full sentences
**Participation**	Plays basketball individually, cannot participate in matches
	Excused school absenteeism (15% of school time)
**Personal factors**	Lower leg amputation due to trauma 6 years ago
	Known as a restless, impulsive teenager
**Environmental factors**	No health insurance
	Parents in conflictual divorce, do not speak Dutch/English

Case 175

Patient (15 years old), known at the community or rehabilitation center where you work, fell down the stairs a week ago. The head wound was sutured. The patient was referred to a neurologist for further examination. No abnormalities were found on the Computed Tomography (CT) scan.<br>Today, the patient presents again at the center.

**Body functions**	Moderate shoulder and neck pain, arm movement partly restricted
	Head wound leaking
**Activities**	Enough energy for daily activities (ADLs)
	Answers questions in full sentences
**Participation**	Recently cut from the U16 basketball team
	Unexcused school absenteeism (30% of school time)
**Personal factors**	Recovered from a broken ankle 2 years ago
	Known as a cheerful, active teenager
**Environmental factors**	Basic health insurance
	Single parent speaks limited Dutch/English

Case 21

Patient (15 years old), known at the community or rehabilitation center where you work, fell down the stairs a week ago. The head wound was sutured. The patient was referred to a neurologist for further examination. No abnormalities were found on the Computed Tomography (CT) scan.<br>Today, the patient presents again at the center.

**Body functions**	Severe shoulder and neck pain, arm movement fully restricted
	Head wound with pus formation
**Activities**	Enough energy for daily activities (ADLs)

	Answers questions in full sentences
**Participation**	Plays basketball as part of a team, participates in matches
	No school absenteeism
**Personal factors**	Complete spinal cord injury (L2) due to bleeding 4 years ago
	Known as a difficult teenager with autistic behaviors
**Environmental factors**	Supplementary health insurance
	Parents speak good Dutch/English

Case 218

Patient (15 years old), known at the community or rehabilitation center where you work, fell down the stairs a week ago. The head wound was sutured. The patient was referred to a neurologist for further examination. No abnormalities were found on the Computed Tomography (CT) scan.
Today, the patient presents again at the center.

**Body functions**	Mild shoulder and neck pain, arm movement not restricted
	Head wound healing well
**Activities**	No energy for daily activities (ADLs)
	Does not answer questions
**Participation**	Recently cut from the U16 basketball team
	Unexcused school absenteeism (30% of school time)
**Personal factors**	Lower leg amputation due to trauma 6 years ago
	Known as a restless, impulsive teenager
**Environmental factors**	Supplementary health insurance
	Parents speak good Dutch/English

Case 60

Patient (15 years old), known at the community or rehabilitation center where you work, fell down the stairs a week ago. The head wound was sutured. The patient was referred to a neurologist for further examination. No abnormalities were found on the Computed Tomography (CT) scan.
Today, the patient presents again at the center.

**Body functions**	Mild shoulder and neck pain, arm movement not restricted
	Head wound healing well
**Activities**	No energy for daily activities (ADLs)
	Does not answer questions
**Participation**	Plays basketball as part of a team, participates in matches
	No school absenteeism
**Personal factors**	Complete spinal cord injury (L2) due to bleeding 4 years ago
	Known as a difficult teenager with autistic behaviors
**Environmental factors**	Basic health insurance
	Single parent speaks limited Dutch/English

Case 171

Patient (15 years old), known at the community or rehabilitation center where you work, fell down the stairs a week ago. The head wound was sutured. The patient was referred to a neurologist for further examination. No abnormalities were found on the Computed Tomography (CT) scan.
Today, the patient presents again at the center.

**Body functions**	Mild shoulder and neck pain, arm movement not restricted

	Head wound healing well
**Activities**	Enough energy for daily activities (ADLs)
	Answers questions in full sentences
**Participation**	Recently cut from the U16 basketball team
	Unexcused school absenteeism (30% of school time)
**Personal factors**	Complete spinal cord injury (L2) due to bleeding 4 years ago
	Known as a difficult teenager with autistic behaviors
**Environmental factors**	No health insurance
	Parents in conflictual divorce, do not speak Dutch/English

Case 131

Patient (15 years old), known at the community or rehabilitation center where you work, fell down the stairs a week ago. The head wound was sutured. The patient was referred to a neurologist for further examination. No abnormalities were found on the Computed Tomography (CT) scan.
Today, the patient presents again at the center.

**Body functions**	Severe shoulder and neck pain, arm movement fully restricted
	Head wound with pus formation
**Activities**	Low energy for daily activities (ADLs)
	Answers questions with yes/no
**Participation**	Plays basketball individually, cannot participate in matches
	Excused school absenteeism (15% of school time)
**Personal factors**	Lower leg amputation due to trauma 6 years ago
	Known as a restless, impulsive teenager
**Environmental factors**	Basic health insurance
	Single parent speaks limited Dutch/English

Case 201

Patient (15 years old), known at the community or rehabilitation center where you work, fell down the stairs a week ago. The head wound was sutured. The patient was referred to a neurologist for further examination. No abnormalities were found on the Computed Tomography (CT) scan.
Today, the patient presents again at the center.

**Body functions**	Moderate shoulder and neck pain, arm movement partly restricted
	Head wound leaking
**Activities**	Low energy for daily activities (ADLs)
	Answers questions with yes/no
**Participation**	Recently cut from the U16 basketball team
	Unexcused school absenteeism (30% of school time)
**Personal factors**	Complete spinal cord injury (L2) due to bleeding 4 years ago
	Known as a difficult teenager with autistic behaviors
**Environmental factors**	Supplementary health insurance
	Parents speak good Dutch/English

Case 241

Patient (15 years old), known at the community or rehabilitation center where you work, fell down the stairs a week ago. The head wound was sutured. The patient was referred to a neurologist for further examination. No abnormalities were found on the Computed Tomography (CT) scan.

Today, the patient presents again at the center.	
**Body functions**	Severe shoulder and neck pain, arm movement fully restricted
	Head wound with pus formation
**Activities**	No energy for daily activities (ADLs)
	Does not answer questions
**Participation**	Recently cut from the U16 basketball team
	Unexcused school absenteeism (30% of school time)
**Personal factors**	Recovered from a broken ankle 2 years ago
	Known as a cheerful, active teenager
**Environmental factors**	No health insurance
	Parents in conflictual divorce, do not speak Dutch/English

Case 153

Patient (15 years old), known at the community or rehabilitation center where you work, fell down the stairs a week ago. The head wound was sutured. The patient was referred to a neurologist for further examination. No abnormalities were found on the Computed Tomography (CT) scan.
Today, the patient presents again at the center.

**Body functions**	Moderate shoulder and neck pain, arm movement partly restricted
	Head wound leaking
**Activities**	No energy for daily activities (ADLs)
	Does not answer questions
**Participation**	Plays basketball individually, cannot participate in matches
	Excused school absenteeism (15% of school time)
**Personal factors**	Complete spinal cord injury (L2) due to bleeding 4 years ago
	Known as a difficult teenager with autistic behaviors
**Environmental factors**	No health insurance
	Parents in conflictual divorce, do not speak Dutch/English

Questions

How do you evaluate the situation of the patient described at this moment?

Very simple (0)	———————•———————	Very complex (100)

Below you will find a list of potential team members for the patient described. In what way should the desired team members collaborate with each other (in writing, through consultation, or in a multidisciplinary team meeting (MDTM)? *One answer per team member is possible. Please select the most ideal option.*

Primary care	**No contact (0)**	**Written (0)**	**Consultation (1)**	**MDTM (1)**
General practitioner	O	O	O	O
Occupational therapist	O	O	O	O
Physical therapist	O	O	O	O
Nurse	O	O	O	O
Nurse practitioner mental health	O	O	O	O
Social worker	O	O	O	O
Another primary professional: [*open text*]	O	O	O	O

Specialized rehab. services	**No contact (0)**	**Written (0)**	**Consultation (1)**	**MDTM (1)**
Pediatric rehabilitation doctor	O	O	O	O
Occupational therapist	O	O	O	O
Physical therapist	O	O	O	O
Speech and language therapist	O	O	O	O
Psychologist	O	O	O	O
Social worker	O	O	O	O
Another rehabilitation professional: [*open text*]	O	O	O	O
**Other setting**	**No contact (0)**	**Written (0)**	**Consultation (1)**	**MDTM (1)**
Youth mental healthcare	O	O	O	O
Child protection	O	O	O	O
School	O	O	O	O
Neurologist	O	O	O	O
Orthopedist	O	O	O	O
Professional in another setting: [*open text*]	O	O	O	O
